# The effect of SUV discretization in quantitative FDG-PET Radiomics: the need for standardized methodology in tumor texture analysis

**DOI:** 10.1038/srep11075

**Published:** 2015-08-05

**Authors:** Ralph T.H. Leijenaar, Georgi Nalbantov, Sara Carvalho, Wouter J.C. van Elmpt, Esther G.C. Troost, Ronald Boellaard, Hugo J.W.L Aerts, Robert J. Gillies, Philippe Lambin

**Affiliations:** 1 Department of Radiation Oncology (MAASTRO), GROW-School for Oncology and Developmental Biology, Maastricht University Medical Centre (MUMC+), Maastricht, the Netherlands; 2 Department of Radiology and Nuclear Medicine, VU University Medical Center, Amsterdam, The Netherlands; 3 Departments of Radiation Oncology and Radiology, Dana-Farber Cancer Institute, Brigham and Women’s Hospital, Harvard Medical School, Boston, MA, USA; 4 Department of Cancer Imaging and Metabolism, H. Lee Moffitt Cancer Center and Research Institute, Tampa, FL, USA

## Abstract

FDG-PET-derived textural features describing intra-tumor heterogeneity are increasingly investigated as imaging biomarkers. As part of the process of quantifying heterogeneity, image intensities (SUVs) are typically resampled into a reduced number of discrete bins. We focused on the implications of the manner in which this discretization is implemented. Two methods were evaluated: (1) R_D_, dividing the SUV range into D equally spaced bins, where the intensity resolution (i.e. bin size) varies per image; and (2) R_B_, maintaining a constant intensity resolution B. Clinical feasibility was assessed on 35 lung cancer patients, imaged before and in the second week of radiotherapy. Forty-four textural features were determined for different D and B for both imaging time points. Feature values depended on the intensity resolution and out of both assessed methods, R_B_ was shown to allow for a meaningful inter- and intra-patient comparison of feature values. Overall, patients ranked differently according to feature values–which was used as a surrogate for textural feature interpretation–between both discretization methods. Our study shows that the manner of SUV discretization has a crucial effect on the resulting textural features and the interpretation thereof, emphasizing the importance of standardized methodology in tumor texture analysis.

In recent years, oncological research has increasingly focused on the prediction of treatment outcome based on individual patient and tumor characteristics^1^, aiming to avoid the one-size-fits-all treatment approach that under- and over-treats a large number of patients. Imaging can play a crucial role here, as it allows for a non-invasive identification and characterization of the tumor^2^,^3^. Positron emission tomography (PET) is a valuable tool for detecting and staging cancer^4^. In recent years, PET imaging has also been increasingly used for decision support^5^, treatment planning^6^,^7^ and response monitoring during radiotherapy^8^. The most widely used PET tracer is [18F] fluoro-2-deoxy-D-glucose (FDG), commonly quantified by standardized uptake values (SUVs)^9^. Easily derived SUV measurements, such as the maximum or peak SUV^10^, are described as predictors for treatment outcome^11^,^12^,^13^,^39^. Additionally, more advanced quantitative imaging features describing tumor image texture (i.e. the spatial arrangement of intensities within the image), which reflect intra-tumor heterogeneity of metabolic activity, are increasingly being investigated as potential imaging biomarkers in lung^14^,^15^, head and neck^16^,^17^, cervical^16^,^18^, esophageal^19^,^20^,^21^ and other cancers^22^,^23^ — a field of research often referred to as ‘Radiomics’^2^,^3^,^24^,^25^,^40^,^41^,^42^.

Efforts have been made to provide guidelines for quality control measures in PET imaging and to standardize patient preparation, dose administration, image acquisition, image reconstruction and SUV normalization, in such a way that absolute SUV measurements are interchangeable in multicenter studies^26^. Interchangeable SUV measurements are very important in PET Radiomics, but the methodology used to determine textural features is also subject to variability. Standardization is therefore needed^27^,^28^,^29^ (Fig. 1).

One important methodological factor is SUV discretization (i.e. resampling image intensity values). Discretization reduces the otherwise infinite possible number of intensity values to a finite set and effectively reduces image noise. Most recent literature describes using a fixed number (e.g. 8, 16) of discrete resampled values or ‘bins’ to divide the image SUV range into equally spaced intervals before calculating textural features^14^,^15^,^16^,^18^,^19^,^20^,^21^,^22^,^28^,^30^,^31^,^32^. Consequently, this results in discretized images with varying bin sizes or ‘intensity resolutions,’ depending on the SUV range. An alternative discretization method is to resample the image SUVs with a fixed bin size in units of SUV (e.g. 0.1, 0.5), maintaining a constant intensity resolution across all tumor images^33^.

When aiming to identify imaging biomarkers in cohort and multicenter studies or trials, it is important that textural features and their ascribed values be directly comparable, both inter- and intra-patient, in order to derive meaningful conclusions. To our knowledge, the effect of the SUV discretization method in this respect has not been previously evaluated and we hypothesize that the aforementioned intensity resolution used for SUV discretization plays a key role in this regard.

The general objectives of our study are to compare both aforementioned conceptually different discretization methods for several popular textural features and to identify which of these methods is most appropriate for texture quantification in a clinical setting. We will specifically investigate the role of the intensity resolution and use a clinical case study to demonstrate the effect of the SUV discretization methodology on the interpretation of the assessed textural features.

## Materials and Methods

### Patients and PET imaging

**Figure 2:**
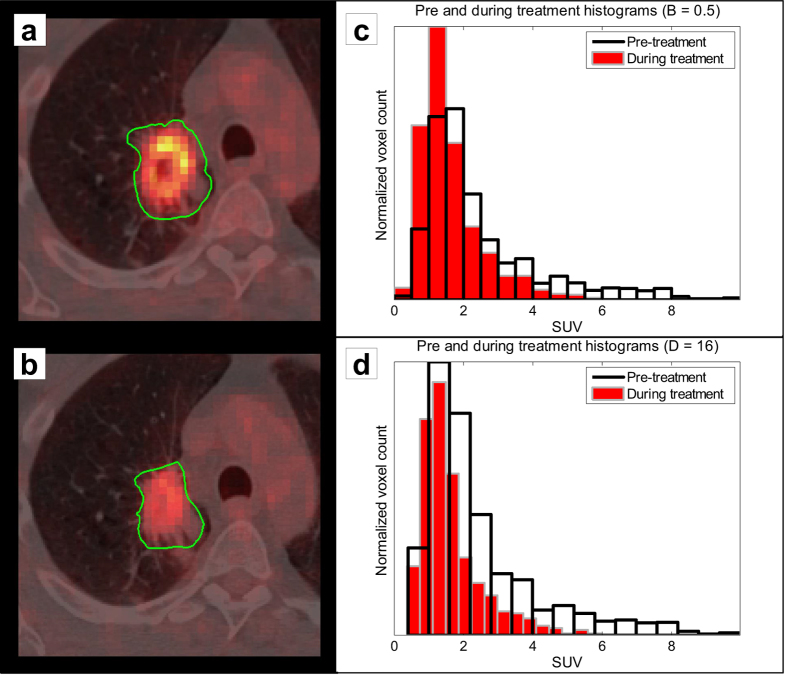
*Left column:* Representative images of sequential imaging for one patient, showing pre-treatment imaging (**a**) and imaging during the second week of radiotherapy (**b**). The tumor delineation is outlined in green. Both images are displayed with the same window/level settings. ***Right column:*** Histograms of the pre-treatment and during treatment images, resampled with a fixed bin size (i.e. intensity resolution) (**c**) or a predefined number of bins (**d**). In (**d**), one can appreciate the difference in resulting intensity resolution when resampling with a fixed number of bins. Pre-treatment and during treatment intensity resolutions were 0.6 and 0.37 [SUV], respectively

This study comprised 35 non-small cell lung cancer (NSCLC) patients who were prospectively included in a clinical trial (NCT00522639) and scheduled for radiotherapy and/or chemotherapy between July and December 2008^11^. 18F-FDG-PET/CT imaging was performed on a Biograph 40 PET/CT scanner (Siemens Medical Solutions) twice: (1) after induction chemotherapy but before radiotherapy and (2) during the second week of radiotherapy (Fig. 2a,b). Patients fasted for at least six hours before imaging. The injected amount of 18F-FDG was (4 × body weight [kg] + 20) MBq. Patients rested 60 minutes before image acquisition. Patients’ blood glucose levels were below 10 mmol/L, so no correction for blood glucose level was applied.

PET images were iteratively reconstructed using normalization- and attenuation-weighted OSEM using 4 iterations, 8 subsets and a 5 mm Gaussian filter. The resulting images had an in-plane pixel size of 4 × 4 mm and a 3 mm slice thickness. PET images were converted into units SUV, normalized by patient body weight^9^. Tumor volumes of interest (VOIs) were manually delineated on fused PET/CT images for treatment planning purposes. Further details are described elsewhere^11^. This study was conducted according to national laws and guidelines and approved by the appropriate local trial committee at Maastricht University Medical Center (MUMC+), Maastricht, The Netherlands. All included patients signed an informed consent form.

### Image processing and feature extraction

SUVs within the VOI were first discretized using: (1) a fixed bin size (B), or intensity resolution, in units of SUV (Fig. 2c) and (2) a fixed number of bins (D), or discrete resampling values (Fig. 2d). For image *I*, let *I*(*x*) represent the SUV of voxel *x, SUV*_*min*_ the minimum SUV in *I* and *SUV*_*max*_ the maximum SUV in *I*. Resampling SUVs into bins with an intensity resolution of B was performed using:
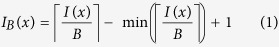
Where term 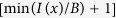 ensures that the bin count starts at 1. We use the shorthand notation R_B_ for this resampling method. Resampling SUVs into D bins was performed using:
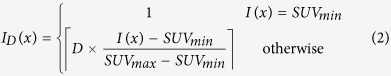
Where the intensity resolution equals 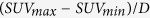. This resampling method is denoted by R_D_. Discretization using R_B_ and R_D_ was performed for different discretization values B (0.05, 0.1, 0.2, 0.5 and 1 [ SUV]) and D (8, 16, 32, 64 and 128), respectively.

Textural features describing the spatial distribution of voxel intensities were calculated from gray-level co-occurrence (GLCM)^34^, gray-level run-length (GLRLM)^35^ and gray-level size-zone texture matrices (GLSZM)^21^. Texture matrices were determined by considering 26 connected voxels (i.e. voxels were considered to be neighbors in all 13 directions in three dimensions) at a distance of 1 voxel. Features derived from GLCM and GLRLM were calculated by averaging their value over all 13 directions. In total, 44 textural features (22 GLCM, 11 GLRLM and 11 GLSZM) were calculated. Changes in feature values between the pre-treatment and during treatment imaging time points were described as delta features, defined as:
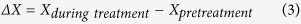


Image analysis was performed in Matlab R2012b (The Mathworks, Natick, MA) using an adapted version of CERR^36^ and software developed in-house to extract textural features. Mathematical definitions for features assessed in this study are described elsewhere^33^.

### Statistical analysis

For both R_B_ and R_D,_ the pairwise intra-class correlation coefficient (ICC)^37^ was calculated for each feature for all possible pairwise combinations of B (ICC_B_) and D (ICC_D_), to assess whether pre-treatment feature values were consistent for different discretization values. The ICC was defined as:
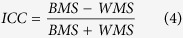
Where BMS and WMS are the between-subjects and within-subjects mean squares, respectively, obtained by Kruskal-Wallis one-way ANOVA. An ICC of 1 indicates perfect agreement (i.e. identical feature values).

Patient rankings according to feature value were created to serve as a surrogate for textural feature interpretation. Pairwise correlations between patient rankings were evaluated with Spearman’s rank correlation coefficient *(ρ)*. We compared patient rakings according to pre-treatment feature values and patient rankings according to delta feature values (Δ*X)*, between all possible pairwise combinations of B (*ρ*^*BB*^), D (*ρ*^*DD*^) and B and D (*ρ*^*BD*^). We considered a pairwise *ρ* to indicate acceptable concordance between rankings when *ρ* >* *0.9. Statistical analysis was performed in Matlab R2012b.

## Results

### Consistency of feature values for varying intensity resolutions

**Figure 3:**
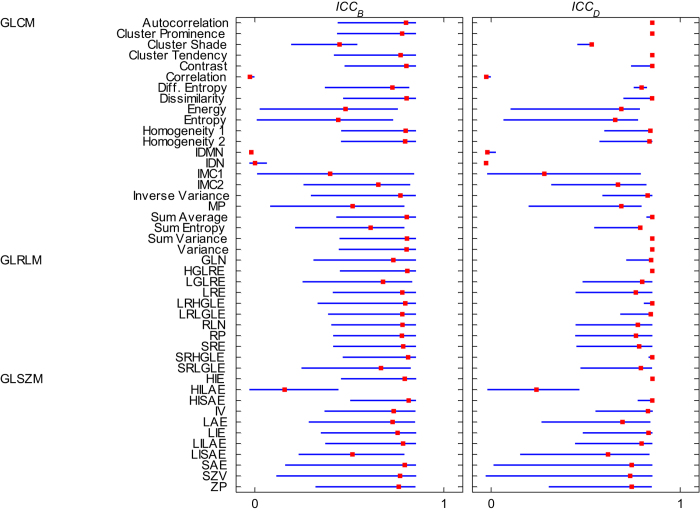
Graphical representation of pairwise ICCs for each feature for different values of B (ICC_B_) and D (ICC_D_), based on pre-treatment imaging. Blue lines extend from the minimum to the maximum observed ICC value. Median ICC values are represented by the red markers. ***Abbreviations of feature groups:***gray-level co-occurrence (GLCM), gray-level run-length (GLRLM) and gray-level size-zone (GLSZM). ***Abbreviations of feature names:*** Difference Entropy (Diff. Entropy), Inverse difference moment normalized (IDMN), Inverse difference normalized (IDN), Informational measure of correlation 1 (IMC1), Informational measure of correlation 2 (IMC2), Maximum probability (MP), Gray-Level Nonuniformity (GLN), High Gray-Level Run Emphasis (HGLRE), Low Gray-Level Run Emphasis (LGLRE), Long Run Emphasis (LRE), Long Run High Gray-Level Emphasis (LRHGLE), Long Run Low Gray-Level Emphasis (LRLGLE), Run-Length Nonuniformity (RLN), Run Percentage (RP), Short Run Emphasis (SRE), Short Run High Gray-Level Emphasis (SRHGLE), Short Run Low Gray-Level Emphasis (SRLGLE), High Intensity Emphasis (HIE), High Intensity Large Area Emphasis (HILAE), High Intensity Small Area Emphasis (HISAE), Intensity Variability (IV), Large Area Emphasis (LAE), Low Intensity Emphasis (LIE), Low Intensity Large Area Emphasis (LILAE), Low Intensity Small Area Emphasis (LISAE), Small Area Emphasis (SAE), Size-Zone Variability (SZV), Zone Percentage (ZP)

To assess whether feature values (using either R_B_ or R_D_) were consistent for different discretization values, we calculated the pairwise ICCs for each feature between different values of B (ICC_B_) and D (ICC_D_), respectively. This analysis was performed on the pre-treatment images. For each feature, we reported the range and median of all pairwise ICCs (Fig. 3). None of the observed pairwise ICCs was higher than 0.85, meaning that textural features and their ascribed value depend on the intensity resolution used for SUV discretization.

### Variability of intensity resolution when resampling with a fixed number of bins

Using R_D_, we determined the pre-treatment and during treatment bin sizes, as well as their difference, for each lesion. We observed a significant variation in both inter- and intra-lesional intensity resolution, which is directly proportional to the SUV range. The ratio of the largest with the smallest observed intensity resolution was 1254% for pre-treatment imaging and 1038% for during treatment imaging. Absolute percentage differences in intensity resolution between pre-treatment and during treatment images ranged between 0.5% and 56%, with a median of 21%.

### Comparing patient rankings based on pre-treatment feature values

**Figure 4:**
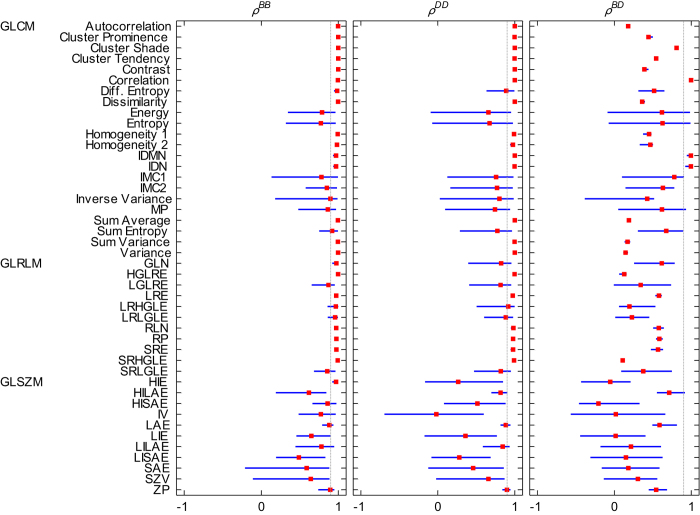
Graphical representation of pairwise Spearman rank correlations between patient rankings according to feature value for different B(*ρ*^BB^), different D (*ρ*^*DD*^) and between different B and D (*ρ*^*BD*^), based on pre-treatment imaging. Blue lines extend from the minimum to the maximum observed pairwise *ρ*. Median *ρ* values are represented by the red markers. The gray vertical line represents *ρ* _=_ 0.9. For abbreviations, see the caption of Fig. 3

For each feature we determined the patient ranking according to feature value, using R_B_ and R_D_ for different resampling values B and D, respectively. We then calculated pairwise *ρ* of patient rankings between different B (*ρ*^*BB*^), different D (*ρ*^*DD*^) and between different B and D (*ρ*^*BD*^). For each feature, we reported the range and median of all pairwise *ρ* (Fig. 4).

We identified 14 GLCM and 6 GLRLM features to give reliable patient rankings for both discretization methods (i.e. all pairwise *ρ*^*BB*^ > 0.9 and all pairwise *ρ*^*DD*^ > 0.9), meaning that patient rankings were nearly not affected by changes in intensity resolution. GLCM ‘Difference entropy,’ GLRLM ‘Gray-Level Non-uniformity (GLN)’ and GLSZM ‘High Intensity Emphasis (HIE)’ were only found to provide robust patient rankings for different resampling values when using R_B_. GLCM features ‘Correlation,’ ‘Inverse Difference Moment Normalized (IDMN)’ and ‘Inverse Difference Normalized (IDN)’ provided very similar patient rankings between both discretization methods, regardless of the value of either B or D (i.e. all pairwise *ρ*^*BD*^ > 0.9). All other features presented dissimilar patient rankings between both discretization methods.

### Comparing patient rankings based on delta feature values

**Figure 5:**
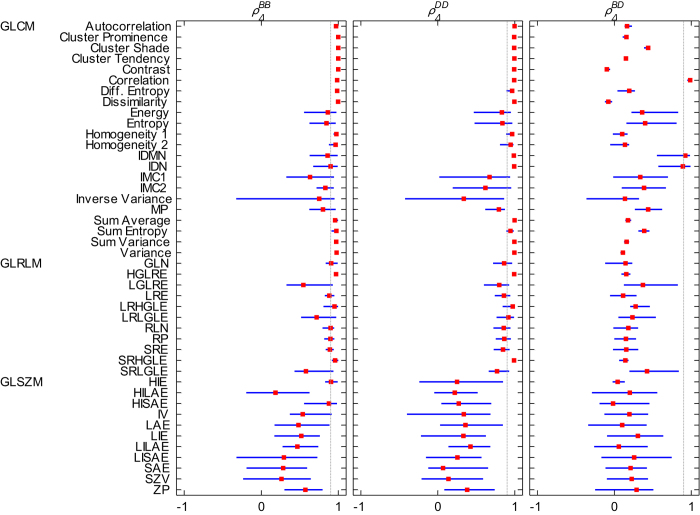
Graphical representation of pairwise Spearman rank correlations between patient rankings according to Δ*X* for different B (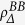), D (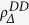), and different B and D(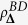). Blue lines extend from the minimum to the maximum observed pairwise *ρ*_**Δ**_. Median *ρ*_**Δ**_ values are represented by the red markers. The gray vertical line represents *ρ* = 0.9. For abbreviations, see the caption of Fig. 3

We also performed pairwise comparisons of patient rankings for each Δ*X* between different B (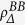), D (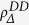) and different B and D (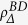). For each feature, we reported the range and median of all pairwise *ρ* (Fig. 5). 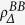 and 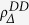 were both higher than 0.9 for of 10 GLCM features and 2 GLRLM features. Δ*X* of GLCM features ‘Difference Entropy,’ ‘Homogeneity 1’ and ‘Sum Entropy’ were only found to give similar patient rankings for different resampling values when using R_B_. For Δ*X* of GLCM features ‘IDMN’ and ‘IDN,’ this was the case when using R_D_ for different D. The high 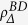 (0.95–1.00) for all pairwise comparisons for GLCM feature ‘Correlation’ indicated highly similar patient rankings based on Δ*X* between both discretization methods, regardless of the value of B or D. Some pairwise 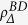 for GLCM features ‘IDMN’ and ‘IDN’ indicated similar patient rankings for Δ*X* as well, but with a large range for 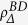 (0.55–0.98 and 0.57–0.99, respectively). For all other Δ*X*, assessed patient rankings were found to be discordant between both discretization methods.

## Discussion

We compared tumor texture analysis based on SUV discretization using either a fixed number of bins (R_D_) or a fixed bin size in units SUV (R_B_), in the context of clinical treatment response assessment. Textural feature values were shown to depend on the intensity resolution used for SUV discretization. Overall, both resampling methods gave discordant results in terms of interpreting textural features. In the following section, we will discuss which method may be appropriate for use in a clinical setting.

### Correct comparison of textural feature values

As pointed out earlier, it is important that textural feature values be directly comparable, both inter- and intra-patient, in order to derive meaningful conclusions from tumor texture analysis. The key role of the intensity resolution in this respect can be illustrated by the mathematical background of histogram bin probabilities. Let *X* be a continuous random variable, such as SUVs in a tumor image, with probability density function *f*(*x*). The bin probabilities *P*(*i*) of the first order histogram, considering equally spaced and non-overlapping bins, are then defined as:
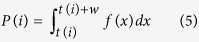
Where 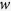 represents the histogram bin size (i.e. the intensity resolution) and *t*(*i*) denotes the left-hand endpoint of bin *i*. Analogous to *P*(*i*), textural matrices are essentially histograms of joint probability densities that describe the probability of a voxel assigned to bin *i* being either (1) adjacent to a voxel assigned to bin *j* (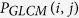), (2) part of a consecutive run of *l* voxels assigned to bin *i* (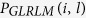) or (3) part of a connected neighborhood of *v* voxels (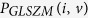).

The aim is to compare textural feature values calculated from these histograms between tumor images. For all tumor images, the image intensities (*x*) are not dimensionless, but measured in SUV units. Maintaining a constant intensity resolution (*w*, in SUV units) across tumor images yields identical histogram probability definitions (*P*(*i*)) for each image, and hence directly comparable numerical values of each calculated feature. Using a non-constant intensity resolution across images causes one to quantify patterns (i.e. texture) on a different intensity scale (in terms of SUV) in each image.

By calculating pairwise ICCs, we observed that feature values indeed depend on the intensity resolution used for SUV discretization (Fig. 3). More importantly, there was a significant inter- and intra-lesional variation in intensity resolution during the course of treatment when using R_D_ for image intensity resampling. Effectively, R_D_ discards the absolute radiotracer uptake information (i.e. metabolic activity), by considering each tumor image to have the same dimensionless range of intensity. In this respect, we consider R_D_ to be a less appropriate choice for SUV discretization in a clinical setting, as it results in textural feature values that are not defined on the same SUV scale for each tumor image. In contrast, a constant intensity resolution is maintained across resampled images when using R_B_ for SUV discretization, which we believe makes it a more suitable method for tumor texture analysis.

### Impact of SUV discretization method and intensity resolution on the interpretation of textural features

Several textural features were found to provide reliable patient rankings using either R_B_ or R_D_ for discretization, suggesting that results based on these features may be compared between studies if they exclusively use R_B_ (where studies may use a different intensity resolution B) or R_D_ (where studies may use a different number of bins D). However, as discussed in the previous section, we find R_D_ to be less appropriate in a clinical setting considering that tumor image intensities are measured in SUV units and that tumor images generally do not have the same SUV range. We therefore illustrated the implications of SUV discretization with R_D_ instead of R_B_ on the interpretation of textural features in our clinical case study. Both discretization methods resulted overall in patients being ranked differently according to their feature value (Figs 4, 5). These results show that the manner of SUV discretization can affect the interpretation of textural features and should therefore be carefully considered in tumor texture analysis.

We furthermore observed that when R_B_ was used, patient rankings for several features were affected by the choice of intensity resolution (B). For those features, at least one pairwise *ρ*^BB^ was found to be lower than 0.9 (Fig. 4). This suggests that results obtained for those features cannot be directly compared when different intensity resolutions are used and also suggests that their interpretation (e.g. prognostic or predictive value) depends on the intensity resolution.

It is noteworthy that the GLCM feature ‘Correlation’ was the only feature observed to have highly similar patient rankings over the course of treatment, regardless of the discretization method or discretization value used (Figs 4, 5). This suggests that results obtained for this particular feature might be reliably compared between studies, provided the same discretization method is used throughout each specific study.

We used different arbitrary values for B and D throughout our study, where we kept the ratio between the smallest and largest B or D approximately the same and reasonably large. Although other values may be used as well, we found this selection to be sufficient to study our objectives. In terms of R_B_ however, an optimal intensity resolution cannot be straightforwardly determined. A value of 0.5 [SUV] has been described earlier, but without substantial motivation^33^. Methods for estimating an optimal intensity resolution could be performed^38^. It should then be emphasized that the same intensity resolution needs to be maintained throughout the entire study, as determining a separate bin size for each individual patient results in non-comparable feature values. However, estimating an optimal intensity resolution does not take into account the aforementioned effect the intensity resolution has on feature interpretation, as well as the fact that using different intensity resolutions may result in complementary information^17^. In this respect, clinical validation including outcome measures is necessary to identify optimal settings that lead to meaningful results in tumor texture analysis.

### Standardization in texture analysis

FDG-PET quantification is affected by several factors, including for instance breathing motion in lung^26^. Recent studies have investigated several technical aspects of FDG-PET-derived textural parameters in different cancer sites, including their test-retest repeatability and robustness regarding tumor delineation or partial volume correction^31^,^32^,^33^, or their variability due to image acquisition and reconstruction parameters^30^. In order to provide a complete overview and acknowledging that feature stability may as well be dependent on the methodology used for SUV discretization in tumor texture analysis, we did not exclude textural features previously reported to have limited repeatability or robustness. Reliability analyses should however be performed at specific settings used in tumor texture analysis, in order to identify those features suitable for treatment assessment. The aforementioned studies point to the importance of robust and standardized PET protocols in terms of reliable quantification of tumor heterogeneity with textural features, especially when the SUV is considered to be an interchangeable quantity^26^,^29^. This becomes even more essential when using fixed intensity resolutions for SUV discretization, as shown in this paper. Our study confirms that using standardized methodology for tumor texture analysis is also an important aspect of identifying and validating imaging biomarkers related to a certain outcome or underlying biology^43^,^44^, between different studies or trials^27^,^28^.

## Conclusion

When aiming to identify and validate imaging biomarkers with tumor texture analysis of FDG-PET, it is important that the textural feature values be directly comparable, both inter- and intra-patient, in order to derive meaningful conclusions. We focused on the effect of SUV discretization and compared tumor texture analysis based on SUV discretization using a fixed intensity resolution (i.e. bin size) in units SUV (R_B_) with using a fixed number of bins (R_D_). We showed that maintaining a constant intensity resolution for SUV discretization across tumor images (R_B_) yields textural feature values that are defined on the same SUV scale, allowing for a meaningful comparison of texture between images. Discretizing SUVs using R_D_ was found to be less appropriate for inter- and intra-patient comparison of textural feature values in a clinical setting. The interpretation of textural features was overall different between both discretization methods and, for several features, affected by the choice of intensity resolution. Our study shows that the manner of SUV discretization has a crucial effect on the resulting textural features and the interpretation thereof and should therefore be carefully considered, underlining the importance of standardized methodology in tumor texture analysis.

## Additional Information

**How to cite this article**: Leijenaar, R. T. H. *et al.* The effect of SUV discretization in quantitative FDG-PET Radiomics: the need for standardized methodology in tumor texture analysis. *Sci. Rep.* **5**, 11075; doi: 10.1038/srep11075 (2015).

## Figures and Tables

**Figure 1:**
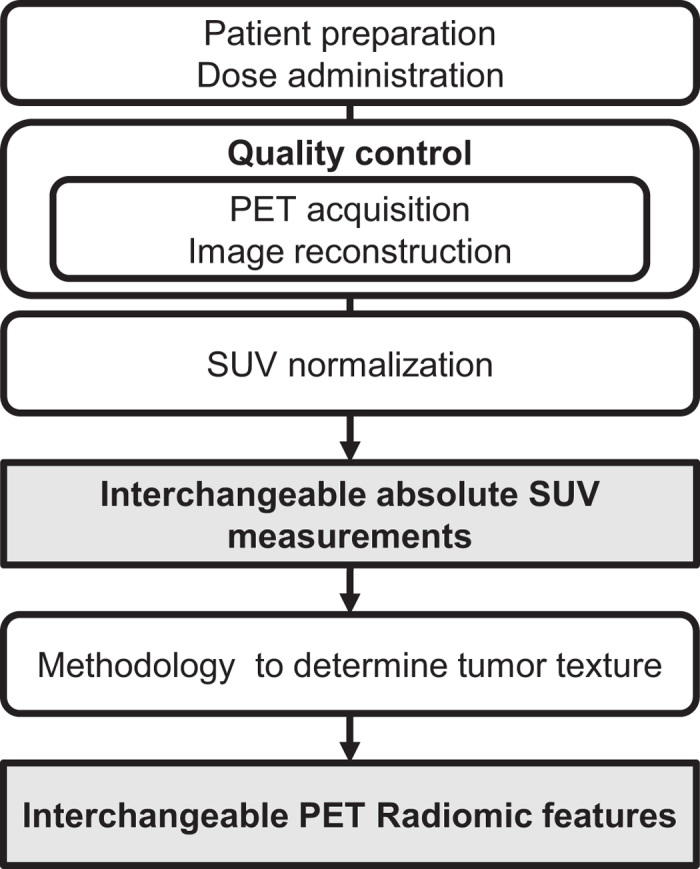
Levels of standardization in PET Radiomics. Interchangeable absolute SUV measurements are obtained by standardizing patient preparation, dose administration, image acquisition, image reconstruction and SUV normalization^26^. Standardization of the methodology used for tumor texture analysis ensures interchangeable PET Radiomic features and their ascribed values
